# Chronic Musculoskeletal Pain, Self-Reported Health and Quality of Life among Older Populations in South Africa and Uganda

**DOI:** 10.3390/ijerph15122806

**Published:** 2018-12-10

**Authors:** Chao Wang, Run Pu, Bishwajit Ghose, Shangfeng Tang

**Affiliations:** 1 School of Public Policy and Management, China University of Mining and Technology, Xuzhou 221116, China; wangchaoccnu@163.com; 2 China National Center for Biotechnology Development, Beijing 100039, China; purun@cncbd.org.cn; 3 Faculty of Social Sciences, School of International Development and Global Studies, University of Ottawa, Ottawa, ON K1N 6N5, Canada; brammaputram@gmail.com; 4 School of Medicine and Health Management, Tongji medical college, Huazhong University of Science and Technology, Wuhan 430030, China

**Keywords:** back pain, chronic musculoskeletal pain, depression, quality of life, older population

## Abstract

Chronic musculoskeletal pain (CMP) is a serious health concern especially among the elderly population and has significant bearing on health and quality of life. Not much is known about the relationship between chronic pain with self-reported health and quality of life among older populations in low-resource settings. Based on sub-national data from South Africa and Uganda, the present study aimed to explore whether the older population living with CMP report health and quality of life differently compared to those with no CMP complaints. This study was based on cross-sectional data on 1495 South African and Ugandan men and women collected from the SAGE Well-Being of Older People Study. Outcome variables were self-reported physical and mental health and quality of life (QoL). Mental health was assessed by self-reported depressive symptoms during the last 12 months. CMP was assessed by self-reported generalised pain as well as back pain. Multivariable logistic regression models were used to measure the association between health and QoL with CMP by adjusting for potential demographic and environmental confounders. The prevalence of poor self-rated health (61.2%, 95% CI = 51.7, 70.0), depression (37.2%, 95% CI = 34.8, 39.6) and QoL (80.5%, 95% CI = 70.8, 87.5) was considerably high in the study population. Mild/moderate and Severe/extreme generalised pain were reported respectively by 34.5% (95% CI = 28.9, 40.5) and 15.7% (95% CI = 12.2, 19.9) of the respondents, while back pain was reported by 53.3% (95% CI = 45.8, 60.4). The prevalence of both types was significantly higher among women than in men (*p* < 0.001). In the multivariate analysis, both generalised pain and back pain significantly predicted poor health, depression and QoL, however, it varied between the two different populations. Back pain was associated with higher odds of poor self-rated health [OR = 1.813, 95% CI = 1.308, 2.512], depression [1.640, 95% CI = 1.425, 3.964] and poor QoL [1.505, 95% CI = 1.028, 2.202] in South Africa, but not in Uganda. Compared to having no generalised pain, having Mild/Moderate [OR = 2.309, 95% CI = 1.219, 7.438] and Severe/Extreme [OR = 2.271, 95% CI = 1.447, 4.143] generalised pain was associated with significantly higher odds of poor self-rated health in South Africa. An overwhelmingly high proportion of the sample population reported poor health, quality of life and depression. Among older individuals, health interventions that address CMP may help promote subjective health and quality and life and improve psychological health.

## 1. Introduction

In the Western countries, non-specific chronic musculoskeletal pain (CMP) represents one of the most common causes of functional disability and of medical visits among adults [1,2,3,4]. Among the elderly, CMP is a major reason for medical consultations, and those living with CMP also report poor physical and psychological health as well as low satisfaction with quality of life [4,5,6]. CMP is an umbrella term and generally encompasses all forms of (non-cancer) musculoskeletal pain e.g., general acute pain and chronic skeletal pain. CMP, especially chronic back pain (CBP), is a major debilitating factor among the elderly populations and has significant bearings on occupational productivity, health and overall well-being [7,8]. CBP is the also the most prevalent form of musculoskeletal condition among adults with the lifetime prevalence being as high as up to 84% [1] and 50% of the patients experiencing more than one episode [9]. Genetic predisposition is generally believed to be a key determinant [1], but back pain has also been reported to have a strong socioeconomic gradient [2,5,10], with the burden being considerably higher among those in lower social strata and engaged in strenuous occupation e.g., heavy physical workload, prolonged lifting and carrying, and frequently twisting or bending [4,5,9,11].

The reason behind the high- and low-income country disparity in research and medical attention on CMP may well be linked to the higher proportion of elderly populations as well as higher prevalence of non-communicable chronic diseases (NCDs) in the HICs. Most LMICs, especially those in Africa, have some of the youngest age structure and a comparatively shorter life expectancy. However, recent population-based studies suggest that LMICs are actually undergoing demographic transitions characterised by increasing life expectancy in parallel with falling fertility and child mortality rates [12,13]. As such, the healthcare challenges due to population aging cannot be ignored, and in some LMICs elderly health issues such as NCDs and CMP have been already been identified as serious public health concerns. In HICs, CBP has been affecting a larger proportion of the elderly population among whom it incurs high direct and indirect costs in terms of health expenditure and loss of productivity [14,15,16]. A review study has reported that CBP costs the United States over $100 billion annually [4] where it is also the second most common cause of disability among the adult population [2].

Apart from the economic costs, CMP has deeper repercussions on quality of life and psychosocial well-being [3,6,17,18]. CMP, as a biological stressor, constitutes a common risk factor for the development of depressive disorders [19]. The pain–depression comorbidity exerts a compounding effect on the progress of each other, with about 85% of patients with CMP likely to be affected by severe depression [6]. Given the biological pathways shared by pain and depression, the presence of one negatively affects the other and has implications for the simultaneous treatment of both [6]. CMP thus acts a serious biological stressor and generally cooccurs with psychological morbidities such as depression, thereby posing significant barriers to coping, management and recovery [17]. The difficulty in effective management of the comorbidity can be particularly challenging in the developing countries like in sub-Saharan Africa owing to fragile mental healthcare system and widespread misconception of mental illness [18,20,21].

Despite the well-documented impacts of CMP on health and quality of life, there has been little research focus on this particular area of public health in Africa [22,23,24]. Therefore, in this study, we aimed to investigate whether CMP has any association with self-reported health (SRH) and QoL among the older population in two African countries including South Africa and Uganda. SRH has been shown to have strong correlation with clinical diagnosis of chronic illnesses and objective measures of morbidity and mortality. The concept of QoL has also been very popular among health researchers as it is regarded as an important psychosocial indicator because it is patient-centered and clinically meaningful [25,26,27]. To this view, we used open-access data on the older population from South Africa and Uganda (>50 years of age) from SAGE Well-Being of Older People Study (WOPS) of World Health Organisation conducted between 2010 and 2013 [28]. The main objective was to investigate whether the older population with CMP complaints report health and quality of life differently compared to those with no such complaints. The data are not recent and therefore may not represent the more recent estimates of SRH and QoL. However, the data provides the opportunity to investigate a research topic that has not been studied in any African setting before. The findings are expected to be useful in the management of CMP and health promotion among the elderly and to be of interest among researcher in the areas of mental health in Africa.

## 2. Materials and Methods

### 2.1. Methods

#### Data Source

For this study, we collected data from SAGE Well-Being of Older People Study (WOPS) of World Health Organization. These were sub-population surveys, and were carried out between 2010 and 2013 in Uganda and South Africa, in partnership with the Medical Research Council/Uganda Virus Research Unit Uganda Research Unit on AIDS, Uganda, and the Africa Centre Demographic Information System (ACDIS) and population-based HIV survey, South Africa [29]. The objectives of these surveys are to provide data on the various health, demographic and social indicators relevant to the well-being and functional status among older people either infected with HIV themselves, or affected by HIV/AIDS in their families. Details of sampling procedures and study protocols were published as WHO reports [30].

### 2.2. Measures

The outcome measures of this study were self-reported health (SRH) and quality of life (QoL). SRH was assessed by two components: (1) physical and (2) psychological. The physical component was assessed by the question: “How satisfied you are with your health?” with the answers ranging from Very Good, Good, Moderate, Bad to Very Bad.

The psychological component was assessed by two questions: “During the last 12 months, have you had a period lasting several days when you felt sad, empty or depressed?”, AND, “During the last 12 months, have you had a period lasting several days when you have been feeling your energy decreased or that you are tired all the time?” with “Yes” and “No” options as answers to both questions. Participants were classified as “Depressed” if they responded “Yes” to any of the questions and “Not depressed” if they responded otherwise. This one-item brief screening scale of lifetime depressive disorders is a commonly used tool in population surveys. The advantages of the brief and self-reported measure is its capacity to capture the overall psychosocial situation from the patient’s perspective and better methodological homogeneity and comparability of the condition of groups across studies and countries [31,32]. However, this relies on the assumption that the symptomatology of a particular disorder (as defined by DSM-IV) will not vary substantially between different countries [31].

QoL was assessed by the question: “How would you rate your overall quality of life?” with the answers being: Very Good, Good, Moderate, Bad, Very Bad. Before the analysis, both SRH and QoL were recategorised as: Good (Very Good, Good), Moderate and Poor/Not-Good (Bad, Very Bad). CMP was measured based on the responses to the following questions on generalised: “During the last 12 months/year have you experienced pain, aching, stiffness or swelling in or around joints (arms, hands, feet) not related to injury & lasted for more than a month?”, AND back pain: “Have you experienced back pain during the last month?”. Both of the questioned were answered as either “Yes” or “No”.

In order to measure the independent association between depression and ADL, the following variables were included as potential confounders based on their known association with the outcome and independent variables: Age (50–59, 60–69, 70–79, 79+ years); Sex (female, male); Current marital status (married, not married); Religion (Christian, Islam/Other); Ever used tobacco (Yes, no); Ever alcohol consumption (Yes, no); Living condition (Very Satisfied, Satisfied, Neither satisfied nor dissatisfied, Dissatisfied, Very dissatisfied); Number of chronic multimorbidity (0, 1, >1); Sleep difficulty (None, Mild, Moderate Severe, Extreme) [5,6,9,10,11,15,16,19,33,34,35]. Chronic multimorbidity [36] was assessed by the self-reported positive diagnosis of the following NCDs: Arthritis, Asthma, Chronic Lung Disease, Diabetes, Cataract, Heart Disease, Stroke, and Hypertension. 

### 2.3. Data Analysis

Datasets were checked for potential outliers and missing values (<0.5%). Next, the datasets were combined to perform pooled analysis. At the first step, we ran a set of descriptive statistics to present sample characteristics and the prevalence of the outcome and independent variables. Given the sociocultural heterogeneity of the sampling populations, we reported the data for South Africa and Uganda separately throughout the analysis. Following that, we ran two sets of multivariate models for each of the outcome variables: one for the South African sample and one for the Ugandan sample. The results of regression analysis were presented as odds ratios and 95% Cis. A two-tailed *p*-value of <0.05 was set as level of significance for all calculations. All analyses were carried out using STATA version 14. 

### 2.4. Ethics Statement

The WOPS survey was approved by the implementing bodies in the respective countries. The datasets were made available in the public data repository of WHO in anonymized form, hence no further approval was necessary for this study.

## 3. Results

### 3.1. Descriptive Statistics

Sample characteristics were presented in Table 1. The majority of the participants were in the youngest age group of 50–59 years (57.5%), female (51.7%), currently not married (79.1%), followers of Christianity (72.6%), never used tobacco (74.3%), and used alcoholic drinks (57.8%). More than half were satisfied with their living condition (58.7%). About one-fifth (20.8%) were living with one NCD and 7.26% with more than one NCDs. Mild/moderate sleep difficulty was reported by 32.2% and severe/extreme by 23.1% respectively.

### 3.2. Prevalence of Generalised Pain and Back Pain, Poor SRH, Depression and Poor QoL

As shown in Table 2, the prevalence of Mild/moderate and Severe/extreme generalised pain was 34.5% (28.9, 40.5) and 15.7% (12.2, 19.9) respectively. The prevalence of both types was significantly higher among women than in men (*p* < 0.001) and among respondents in South Africa than for Uganda.

As shown in Table 3, overall more than half of the respondents reported having back pain (53.3%, 95% CI = 45.8, 60.4). Similar to generalised pain, the prevalence of back pain was significantly higher among women than in men (*p* < 0.001). However, unlike for generalised pain, the prevalence of back pain was higher for Uganda (*p* < 0.001).

Comparative prevalence of the outcome variables (SRH, depression, QoL) was presented in Table 4. Poor SRH and QoL were reported by respectively 61.2% (51.7, 70.0) and 80.5% (70.8, 87.5), and depression by 37.2% (34.8, 39.6) of the respondents. The prevalence of poor health (66.5% vs. 55.3%) and QoL (81.3% vs. 79.6%) was higher among women, whereas that of depression was higher among men than in women (40.0% vs. 34.6%). At country level, South Africa had a higher prevalence of depression and poor QoL, whereas Uganda had higher prevalence of poor SRH. 

### 3.3. Multivariable Analysis

The predictors of poor SRH, depression and Quality of life measured with multivariate analysis were summarised in Table 5. Being in the higher age showed a protective effect against poor SRH in South Africa. Those in the age group of 70–79 years and 80+ years had respectively 0.431 (0.278, 0.669) and 0.380 (0.198, 0.727) times lower odds of reporting poor SRH in South Africa. Compared with those in the lowest age group (50–59 years), the odds of poor QoL among the 70–79 years old were respectively 0.326 (0.188, 0.568) and 0.369 [0.159, 0.858] times lower in South Africa and Uganda. Sex and current marital status did not appear to have any significant association with any of the outcome measures. In South Africa, having Islam/other religious affiliation showed lower odds of reporting depression (OR = 0.564, 95% CI = 0.322, 0.989). Satisfaction with living conditions was found to be protective against poor QoL in South Africa (OR = 0.493, 95% CI = 0.277, 0.877) and Uganda (OR = 0.333, 95% CI = 0.148, 0.749). Compared with having severe/extreme sleep difficulty, having mild/moderate sleep difficulty reduced the odds of poor SRH for South Africa and depression and poor QoL for both South Africa and Uganda. Having generalised pain and back pain also showed strong negative associations with depression. For example, compared to having no generalised pain, having Mild/Moderate (OR = 2.309, 95% CI = 1.219, 7.438) and Severe/Extreme (OR = 2.271, 95% CI = 1.447, 4.143) generalised pain was associated with significantly higher odds of poor SRH in South Africa. Having back pain also increased the odds of poor SRH (OR = 1.813, 95% CI = 1.308, 2.512), depression (1.640, 95% CI = 1.425, 3.964) and poor QoL (1.505, 95% CI = 1.028, 2.202) in South Africa, but not in Uganda.

## 4. Discussion

Healthcare systems in sub-Saharan Africa have been struggling to promote population health and quality of life for the ever-growing population. The challenges stemming from poor health issues on socioeconomic development are likely to become even more pronounced due to population aging and the unpreparedness of healthcare systems to meet the medical needs unique to the older population’s health. Research evidence is necessary to inform the health policy makers regarding the distribution of health problems across the different sociodemographic groups. To date, little is known regarding the predictors of poor health and quality of life among the older population in Africa. Therefore, in this study, we presented a quantitative analysis of physical and mental health status among older individuals in South Africa (per capita GDP of 7725 USD as of 2010) and Uganda (595 USD as of 2010), which were respectively categorised as low- and upper-middle income countries by the World Bank during the period of conducting the surveys (2010–2013). This stark inequality in per capita income is reflected through life expectancy as well (59.89 years in Uganda vs. 62.77 years South Africa as of 2016). We found a considerably high prevalence of poor self-reported health, quality of life and depression in both of the countries. Contrary to intuition, South Africa was found have a higher prevalence of depression and poor QoL than in Uganda. However, Uganda had a higher prevalence of poor SRH with more than two-thirds of the respondents reporting their health status as not good. 

The high prevalence of poor SRH reflects an urgent need for a focus shift to geriatric health management/promotion in these countries. So far, the majority of the countries in sub-Saharan Africa have made appreciable progress in reducing the rates of malnutrition and maternal and child mortality rates. In contrast to the health status of children and adults, the health and social needs of the elderly populations have received very limited attention so far. With rising life expectancy and the growing proportion of elderly populations, the healthcare systems should make necessary strategies to restructure the care delivery model to make sure the health needs of the vulnerable demographic segments, such as the elderly, are not left unmet.

There was also a strong gendered pattern in the prevalence of poor health and depression such that the prevalence of poor health and quality of life was reported by a significantly higher proportion of among women, whereas that of depression was higher among men. That women generally report poorer psychological health compared with men has been highlighted by previous researchers in developed countries [37,38,39]. However, the gendered nuances in the prevalence of depression between developed and developing countries remains to be explored. The literature review suggests that women’s psychosocial health is another underappreciated area in health research in Africa. Previous studies have reported inequalities in healthcare utilisation among women in several African countries [40,41]. Our findings add to the current literature that women may also experience poorer health and quality of life than men, and call for further research to investigate the disparities in health outcomes between men and women in this region.

Apart from the high prevalence of poor health and quality of life, our findings also indicate significant association between quality of life with environmental and chronic conditions such as generalised and back pain. Satisfaction with the living environment independently predicted poor quality of life in both populations. A poor living environment can exert negative effects on the overall physical and psychosocial health of individuals through complex mechanisms. For instance, lack of access to improved water, sanitation and hygiene (WASH) is associated with frequent bouts of infectious diseases among both children and adults [42,43,44]. Environmental stressors such as insalubrious housing, ambient air pollution, and noisy and congested living places can compromise health outcomes through affecting sleep and heightened stress by hindering the adoption of healthy lifestyles particularly among the elderly because of the diminishing immunity and functional capabilities [45,46]. These findings highlight the need for paying attention to the living environment among the older population with an aim to promote perceived health and psychosocial conditions. Old age is also associated with higher prevalence of chronic-type illness that requires life-long treatment and behavioral management. Therefore, investing in the living environment can be an important strategy not only for addressing general health complaints, but also for better management of chronic conditions for the growing elderly population in Africa.

The findings also indicated that suffering from generalised pain and back pain can significantly reduce health and QoL and increase perceived depression in both countries. Physical pain of any sort is a strong biological stressor, and its mechanism is closely interlinked with the pathophysiology of depression. Sleep quality itself can serve as an indicator of health and quality of life measures. Thus, experiencing physical pain can also affect health and quality of life through exacerbating mental health which itself is a key predictor of both health and QoL outcomes. Despite these interesting findings, we are unable to assess the causality or directionality of the associations as the data were cross-sectional. However, the growing evidence on the negative effect of pain on poor mental health status might be indicative of a strong causal relationship. So far, not much is known about the association in developing countries. The present study is therefore expected to be a good contribution to the literature and provides encouragement for more in-depth studies in this field. 

Given the growing number of aged populations with a concomitant rise in NCDs such as diabetes mellitus, hypertension and obesity, large-scale and cross-country studies on health status among the elderly population represent an urgent imperative. This is particularly so in sub-Saharan Africa owing to the fragile healthcare systems that are filing to meet the health needs of the vulnerable population. In the current literature, there is insufficient evidence regarding perceived health and QoL among the elderly population in African countries. Hence, the findings of the present study can be extremely insightful for health practitioners as well as geriatric health researchers in South Africa, Uganda and in other countries across the continent. Apart from the important contributions, there are several limitations to report. As mentioned earlier, the surveys were cross-sectional and therefore cannot indicate any causality between the outcome and predictor variables. The sample population was not representative and subject to selection bias, and hence the results are not generalizable for the entire elderly population in the countries being studied. Last but not least, the variables were self-reported and thereby remain subject to recall and reporting bias.

## 5. Conclusions

In conclusion, we found a considerably high prevalence of poor self-reported quality of life and depression among older men and women in South Africa and Uganda, with the overall prevalence of depression being higher among men compared with women. The findings suggest that improving the situation of the living environment and addressing generalised pain and back pain may help improve subjective health and quality of life and promote mental well-being in these populations. However, healthcare systems in African countries are largely underequipped to meet the medical needs of older populations. Further research evidence is necessary to inform the health policy makers regarding the risk factors of poor health outcomes among the elderly population and effective intervention techniques to address them.

## Figures and Tables

**Table 1:** Participant characteristics. SAGE WOPS 2010–2013.

	Description	N = 1495	Percentage
Age groups			
50–59	Current age of the participants	860	57.5
60–69		301	20.1
70–79		232	15.5
80+		102	6.8
Sex			
Male	Sexual orientation	722	48.3
Female		773	51.7
Marital status			
Not Married	Current living arrangement	1183	79.1
Married/Cohabitating		312	20.9
Religion			
Catholic	Religious affiliation	1086	72.6
Islam/Other		409	27.4
Living condition			
Satisfactory	Self-reported situation of living environment	870	58.7
Neural		426	28.5
Not Satisfactory		199	12.8
Tobacco			
Yes	History of tobacco use	387	25.7
No		1108	74.3
Alcohol			
Yes	History of alcohol use	865	57.8
No		629	42.2
Sleep difficulty			
None	Self-reported difficulty in falling asleep	669	44.7
Mild/Moderate		481	32.2
Severe/Extreme		345	23.1
Multimorbidity			
0	Total number of diagnosed NCDs	1070	71.6
1		311	20.8
>1		114	7.6
Country			
South Africa	Country of survey	514	34.4
Uganda		981	65.6

N.B. NCDs = Noncommunicable chronic diseases. WOPS = SAGE Well-being of Older People Study.

**Table 2:** Prevalence of generalised pain among older men and women in South Africa and Uganda.

	None 49.7% (41.4, 58.1)	Mild/Moderate 34.5% (28.9, 40.5)	Severe/Extreme 15.7% (12.2, 19.9)	*p*
Sex				
Male	63.8% (53.7, 72.7)	24.4% (18.3, 31.8)	11.7% (8.2, 16.4)	<0.001
Female	37.2% (30.9, 43.7)	43.5% (38.6, 48.6)	19.3% (15.8, 23.3)	
Country				
South Africa	27.4% (22.0, 33.4)	49.9% (45.0, 54.9)	22.7% (19.0, 26.9)	<0.001
Uganda	61.3% (54.6, 67.6)	28.4% (23.6, 33.7)	10.4% (7.9, 13.5)	

**Table 3:** Prevalence of back pain among older men and women in South Africa and Uganda.

	Has Back Pain 53.3% (45.8, 60.4)	No Back Pain 46.7% (39.5, 54.0)	*p*
Sex			
Male	43.5% (35.7, 51.6)	62.2% (55.4, 67.8)	<0.001
Female	56.5% (48.4, 64.3)	37.8% (32.0, 44.1)	
Country			
South Africa	27.4% (18.9, 37.6)	72.6% (62.2, 80.9)	<0.001
Uganda	64.7% (60.1, 69.1)	35.2% (30.7, 39.6)	

**Table 4:** Differential prevalence in physical and cognitive ADL difficulties by sex and country.

	Poor SRH 61.2% (51.7, 70.0)	Depression 37.2% (34.8, 39.6)	Poor QoL 80.5% (70.8, 87.5)
Sex			
Male	55.3% (44.4, 65.8)	40.0% (36.1, 44.1)	79.6% (67.5, 88.0)
Female	66.5% (58.3, 73.9)	34.6% (30.5, 39.0)	81.3% (72.7, 87.6)
*p*-value	<0.001	<0.001	<0.001
Country			
South Africa	46.5% (32.7, 60.9)	69.8% (59.2, 78.7)	82.1% (67.8, 90.9)
Uganda	67.8% (61.6, 73.5)	22.5% (17.9, 28.0)	79.8% (71.1, 86.4)
*p*-value	<0.001	<0.001	<0.001

**Table 5:** Association between CMP with SRH, depression and QoL in South Africa and Uganda.

	South Africa			Uganda		
	SRH	Depression	Quality of Life	SRH	Depression	Quality of Life
Age (50–59)						
60–69	0.838	0.943	0.834	1.174	1.226	0.921
	(0.584, 1.203)	(0.616, 1.445)	(0.559, 1.246)	(0.655, 2.106)	(0.482, 3.117)	(0.512, 1.658)
70–79	0.431 ***	0.618	0.326 ***	0.582	0.523	0.369 *
	(0.278, 0.669)	(0.376, 1.017)	(0.188, 0.568)	(0.278, 1.218)	(0.127, 2.158)	(0.159, 0.858)
80+	0.380 **	0.473 *	0.469	0.396	0.475	1.112
	(0.198, 0.727)	(0.250, 0.896)	(0.218, 1.008)	(0.0800, 1.956)	(0.0378, 5.976)	(0.230, 5.380)
Sex (Male)						
Female	1.106	1.024	1.195	1.497	0.451	0.964
	(0.738, 1.656)	(0.650, 1.612)	(0.752, 1.897)	(0.776, 2.886)	(0.156, 1.309)	(0.492, 1.890)
Currently married (No)						
Yes	1.154	0.700	1.091	1.043	0.797	0.725
	(0.786, 1.693)	(0.442, 1.109)	(0.704, 1.689)	(0.570, 1.906)	(0.291, 2.179)	(0.381, 1.378)
Religion (Christian)						
Islam/other	1.161	0.564 *	0.964	0.965	0.144	0.880
	(0.724, 1.861)	(0.322, 0.989)	(0.557, 1.669)	(0.427, 2.181)	(0.0152, 1.355)	(0.385, 2.013)
Smokes (No)						
Yes	0.995	1.061	0.803	1.246	0.607	1.225
	(0.690,1.435)	(0.708,1.591)	(0.531,1.216)	(0.599,2.593)	(0.161,2.293)	(0.584,2.570)
Alcohol (No)						
Yes	0.936	1.081	1.122	0.807	2.493	0.918
	(0.619, 1.416)	(0.689, 1.697)	(0.697, 1.807)	(0.445, 1.465)	(0.929, 6.692)	(0.496, 1.701)
Living condition (Not satisfactory)						
Neutral	0.776	0.345 ***	0.205 ***	0.730	0.663	0.169 ***
	(0.538, 1.120)	(0.217, 0.550)	(0.125, 0.337)	(0.408, 1.308)	(0.233, 1.890)	(0.0884, 0.323)
Satisfactory	1.378	0.895	0.493 *	1.025	3.013	0.333 **
	(0.856, 2.218)	(0.529, 1.514)	(0.277, 0.877)	(0.463, 2.268)	(0.910, 9.980)	(0.148, 0.749)
Sleep difficulty (Severe/Extreme)						
Mild/Moderate	0.573 **	2.104 ***	0.356 ***	0.819	3.366 *	0.346 **
	(0.404, 0.813)	(1.388,3.189)	(0.233, 0.544)	(0.440, 1.524)	(1.295, 8.747)	(0.174, 0.686)
None	0.483 **	2.380 ***	0.487 *	0.520	3.149	0.649
	(0.300, 0.777)	(1.501, 3.775)	(0.280, 0.849)	(0.230, 1.177)	(0.954, 10.39)	(0.273, 1.545)
Multimorbidity (0)						
1	0.745	1.027	0.879	0.640	1.035	0.735
	(0.526, 1.056)	(0.695, 1.517)	(0.589, 1.310)	(0.373, 1.096)	(0.427, 2.507)	(0.416, 1.297)
>1	0.735	1.426	0.936	2.263	1.567	5.955 *
	(0.429, 1.259)	(0.833, 2.441)	(0.504, 1.736)	(0.497, 10.31)	(0.245, 10.03)	(1.316, 26.94)
General pain (No)						
Mild/Moderate	2.309 ***	1.496	0.756	0.944	3.118 *	0.900
	(1.219,7.438)	(0.938,2.388)	(0.378, 1.818)	(0.496, 2.606)	(1.175,8.270)	(0.498, 1.624)
Severe/Extreme	2.271 ***	3.482 ***	0.655	0.709	13.86 ***	0.856
	(1.447, 4.143)	(2.060, 5.885)	(0.371, 2.158)	(0.267, 1.571)	(3.700, 21.88)	(0.383, 2.638)
Back pain (No)						
Yes	1.813 ***	1.640 *	1.505 *	1.538	0.581	1.210
	(1.308,2.512)	(1.425, 3.964)	(1.028, 2.202)	(0.899, 2.629)	(0.244, 1.382)	(0.685, 2.139)

N.B. Figures represent Odds ratios with95% confidence intervals in () brackets; reference categories in () brackets. * *p* < 0.05, ** *p* < 0.01, *** *p* < 0.001.
